# Dynamic of Immune Response induced in Hepatitis B Surface Antigen-transgenic Mice Immunized with a Novel Therapeutic Formulation

**DOI:** 10.5005/jp-journals-10018-1161

**Published:** 2016-07-09

**Authors:** Freya M Freyre Almeida, Aracelys Blanco, Heidy Trujillo, Dunia Hernández, Daymir García, José S Alba, Matilde López Abad, Nelson Merino, Yadira Lobaina, Julio C Aguilar Rubido

**Affiliations:** 1 Vaccine Division, Center for Genetic Engineering and Biotechnology, Havana, Cuba; 2 Animal Facilities, Center for Genetic Engineering and Biotechnology, Havana, Cuba; 3 Technology Development Division, Center for Genetic Engineering and Biotechnology, Havana, Cuba; 4 Food and Pharmacy Faculty, University of Havana, Havana, Cuba

**Keywords:** Chronic hepatitis B, HBcAg, HBsAg, HBV transgenic mice, Therapeutic vaccine.

## Abstract

The development of therapeutic vaccines against chronic hepatitis B requires the capacity of the formulation to subvert a tolerated immune response as well as the evaluation of histopathological damage resulting from the treatment. In the present study, the dynamicity of induced immune response to hepatitis B surface antigen (HBsAg) was evaluated in transgenic mice that constitutively express the HBsAg gene (HBsAg-tg mice). After immunization with a vaccine candidate containing both surface (HBsAg) and core (HBcAg) antigens of hepatitis B virus (HBV), the effect of vaccination on clearance of circulating HBsAg and the potential histological alterations were examined. Transgenic (tg) and non-transgenic (Ntg) mice were immunized by intranasal (IN) and subcutaneous (SC) routes simultaneously. A control group received phosphate-buffered saline (PBS) by IN route and aluminum by SC route. Positive responses, at both humoral and cellular levels, were obtained after five immunizations in HBsAg-tg mice. Such responses were delayed and of lower intensity in tg mice, compared to vaccinated Ntg mice. Serum IgG response was characterized by a similar IgG subclass pattern. Even when HBsAg-specific CD8^+^ T cell responses were clearly detectable by gamma-interferon ELISPOT assay, histopathological alterations were not detected in any organ, including the liver and kidneys.

Our study demonstrated, that it is possible to subvert the immune tolerance against HBsAg in tg mice, opening a window for new studies to optimize the schedule, dose, and formulation to improve the immune response to the therapeutic vaccine candidate. These results can be considered a safety proof to support clinical developments for the formulation under study.

**How to cite this article:**

Freyre FM, Blanco A, Trujillo H, Hernández D, García D, Alba JS, Lopez M, Merino N, Lobaina Y, Aguilar JC. Dynamic of Immune Response induced in Hepatitis B Surface Antigen-transgenic Mice Immunized with a Novel Therapeutic Formulation. Euroasian J Hepato-Gastroenterol 2016;6(1):25-30.

## INTRODUCTION

Hepatitis B virus (HBV) infection is still a major public health problem worldwide. Most people develop acute hepatitis, which is controlled by both humoral and cellular immune responses following acute infection.^1,2^ However, around 2 to 20% of infected adults and 95% of infected newborns in HBV-endemic areas fail to resolve the infection and become chronic carriers.^3,4^ These individuals that remain persistently infected with HBV develop a weak and antigenically restricted antiviral immune response responsible for a chronic necroinflammatory disease. A considerable number of HBV carriers progress to more severe complications like cirrhosis and hepatocellular carcinoma.^5-7^ On the contrary, current therapeutic approaches to control chronic HBV-associated hepatitis are unsatisfactory, because of its partial efficacy, high cost, and important side effects.^8-10^

Clinical and experimental findings indicate that immune-mediated clearance mechanisms can be spontaneously activated or induced, even in chronic hepatitis B.^11-13^

Studies conducted in mice expressing the HBV proteins at different levels have shown the immunotherapeutic potentialities of protein- and DNA-based vaccines for the treatment of chronic hepatitis B–infected patients.^10^ The Center for Genetic Engineering and Biotechnology (CIGB) of Havana, Cuba, has developed a vaccine candidate based on the combination of surface and nucleocapsid antigens of HBV.^14,15^ Immunizations of Balb/c mice with this vaccine candidate enhanced both humoral and cellular immune responses against both HBsAg and HBcAg proteins after administration by mucosal and parenteral routes.^16^

The present work evaluated the ability of simultaneous co-administration, by nasal and SC routes of the vaccine candidate based in surface and nucleocapsid viral antigens to induce HBsAg-specific humoral and cellular responses in HBsAg-transgenic (HBsAg-tg) mice.^17^ It also evaluated the functionality of such induced auto-antibodies to neutralize the HBsAg from the circulation of transgenic mice and the immunopathological risk derived from specific immune response in such experimental conditions.

## MATERIALS AND METHODS

### Animals

Female, adult Balb/c (H-2^d^ haplotype), and HBsAg-tg mice (with Balb/c genetic background) were kept under controlled conditions and certified room in the animal facility at CIGB, Havana, Cuba. Balb/c mice were purchased from the National Center for Animal Breeding (CENPALAB, Havana, Cuba). HBsAg-tg mice expressing high levels of HBsAg in circulation (adw2 serotype) and in several other organs and tissues were produced at CIGB, Havana, Cuba.^17,18^

Mice were used in this study after the institutional review board approval and care were in compliance with recommendations of the Guide for Care and Use of Laboratory Animals of CIGB, Havana, Cuba.

### Formulations used for Vaccination

The combined HBs/HBc formulation consists of 0.1 mg/mL of each recombinant HBsAg and HBcAg antigens, in phosphate buffer/EDTA. For SC injection, a formulation containing 5 µg of each antigens and 0.5 mg/mL of aluminum hydroxide [Al(OH)_3_] (Brenntag Biosector, Denmark) per dose was used.

### Immunization Schedule

HBsAg-transgenic and non-transgenic (Ntg) mice were randomly assigned to four groups of treatments, each group with 11 and 9 experimental units of HBsAg-tg and Ntg mice respectively. Animals were immunized 10-fold by the IN and SC routes at 2 weeks’ interval between inoculations. The groups, immunogens, doses, administration routes, and the number of animals per group are shown in Table 1. Serum samples were repeatedly obtained from individual mice, vaccine immunized or placebo inoculated control mice by retro-orbital bleeding procedure at different time points post injection. Sample were processed and preserved at –20°C for further serological examination.

**Table 1:** **Table 1:** The immunization groups, immunogens, routes, doses, and numbers of animals per group used in the study are included. The immunizations were carried out 10 times every 2 weeks

*Group*		*Experimental* * unit ^a^*		*Immunogen*		*Route*		*Dose * *(μl)*		*No.* * of mice*	
1		HBsAg-tg		PBS		IN		50		11	
				PBS + Al(OH)_3_		SC		100			
2		HBsAg-tg		HBs/HBc		IN		50		11	
				HBs/HBc + Al(OH)_3_		SC		100			
3		Ntg		PBS		IN		50		9	
				PBS + Al(OH)_3_		SC		100			
4		Ntg		HBs/HBc		IN		50		9	
				HBs/HBc + Al(OH)3		SC		100			

^a^All animals used in this study were female and with Balb/c genetic background (H-2d haplotype)

Ten days after the 3rd (day 38), 5th (day 66), and 10th (day 136) doses, spleens from three animals per group were removed and cells were isolated for *in vitro* cellular assays (Table 1).

### Determination of Serum Antibody Levels

Indirect ELISA was used to measure the HBsAg-specific antibody levels, as previously described.^16^ Briefly, the micro-ELISA plate (Costar, High Binding, USA) were coated with recombinant HBsAg (adw-2 subtype) at a concentration of 5 µg/mL in 0.1 M sodium carbonate buffer (ph 9.6) for 16 hours at 4°C. After blocking the sera samples diluted in sample buffer, PBS/1% dry milk and 1% Tween 20 were added to the Ag-coated wells. For IgG isotype-specific detection, HRP-conjugated rabbit antimouse IgG1 and IgG2a antibodies (ICN Biomedicals, USA) were used. Finally the plates were washed fivefold and the o-phenylenediamine (Sigma, St Louis, USA)/hydrogen peroxide substrate solution was applied. After 15 minutes the reaction was stopped with 2.8 M sulphuric acid solution and the plates were read at 492 nm wavelength with a Multiskan Sensident (LabSystem, Finland) reader. The cut-off value of the assay was defined as twice the optical density (OD) value of negative control serum. The sample was considered positive if the mean value of optical density of two determinations was equal to or above the cut-off value of the assay. To determine the serum Ab titers a standard hiperimmune serum was used and data processed by an Excel program.

### *In vitro* Re-stimulation of CD8+ T-Cells

Ten days after the 3rd, 5^th^, and 10th immunization, spleens were removed and single-cell suspensions were isolated after erythrocytes lyses. Cells were washed several times, resuspended in RPMI 1640 (Gibco, USA) complete medium [supplemented with 10% fetal bovine serum (FBS; PAA, Canada), 2 mM glutamine, 2 mM sodium pyruvate, and antibiotics], and counted. After several washes, cells were counted and distributed in 25 cm^2^ culture flasks at 2 × 10^6^ cells/mL and stimulated with 10 µg/mL of HBsAg-S_28-39_ peptide (sequence IPQSLDSWWTSL). After being cultured for 4 days, one half of the culture medium was substituted with fresh medium to which 20 U/mL recombinant hu IL-2 was added. On day 7th cells were collected and counted.

### Antigen-presenting Cell Preparations

For CD8+ T-cell response assessment, p815 mastocytoma cell line was used as the target cell. Cells were incubated in complete medium with 10 µM S_28-39_ peptide for 1 hour at 37°C in a 5% CO_2_ atmosphere. After incubation p815 cells were further incubated for 15 minutes with mitomycin C (Sigma, USA), washed extensively to eliminate mitomycin C remains, and counted. In parallel, p815 cells received the same treatment but without peptide pulsing, to be used as negative controls.

### Study of CD8+ Gamma-Interferon (IFN)-Secreting Cells by ELISPOT

Microplate with mixed cellulose ester membrane (Millipore, Bedford, MA, USA) were coated with 5 µg/mL of anti-gamma-IFN mAb R46A2 (BD Pharmingen) and incubated overnight at 4°C. Plates were washed three times and blocked with RPMI 1640 containing 10% FBS for 1 hour at 37°C. Splenocytes, previously re-stimulated with the peptide S_28-39_, were plated out at varying densities (5 × 10^5^, 1 × 10^5^, 0.5 × 10^5^ cells/mL) and stimulated for 20 hours with 1 × 10^5^ peptide-loaded p815 cells at 37°C in a 5% CO_2_ atmosphere. Unpulsed P815 cells were used as negative controls. As positive controls, splenocytes incubated with 2.5 µg/mL of Concanavalin A (ConA) (Sigma, USA) were used. The plates were washed extensively and spots visualized using 0.5 µg/mL biotin-conjugated anti-gamma IFN mAb XMG1.2 (BD Pharmingen, USA) for 2 hours at room temperature and for 1 hour with a peroxidase-labeled streptavidin (Amersham Pharmacia Biotech, UK). Finally, the plates were washed with 0.05% Tween 20 in PBS, and afterward with PBS, and the spots revealed by adding 100 μl of 3-amino-9-ethylcarbazole (AEC, Sigma, USA) solution. After 15 minutes of incubation with the substrate, the plates were washed with tap water, dried, and counted using a stereomicroscope. Results were expressed in terms of the number of gamma-IFN-secreting cells per 10^6^ splenocytes after substracting mean counts from the negative wells. Determinations are considered positive if their mean values exceed in twice the mean count of the negative control group and, in addition, the difference in the mean count number should be at least 10 counts obtained by direct reading of the plates. Positive criterion: Y ≥ 2X + 10, where Y is the number of spots in the wells with P815 loaded with S_28-39_ and X is the number of spots for unpulsed P815.

### Histopathological Studies

For each group (4 HBsAg-tg and 3 Ntg), the liver, kidneys, heart, and lungs were macroscopically inspected and lesions scored under magnification. Further comparison to control animals was carried out. Tissues samples were immediately fixed by immersion in 10% (v/v) buffered formalin and routinely embedded in paraffin. Sections were cut at 5 µm and stained with hematoxylin and eosin (H & E).

### Statistical Analysis

The results of the ELISA and ELISPOT experiments were processed using version 4.00 for Windows, of GraphPad Prism Software (San Diego, CA, USA). Unless noted, data are presented as geometric mean ± SD. Comparisons between the two groups were performed by two-tailed Student’s t-test. The effect of treatment in the HBsAg levels in serum was evaluated by a paired student’s t-test. A p-value < 0.05 was considered statistically significant.

## RESULTS

### Coadministration of HBsAg and HBcAg Antigens induced HBsAg-specific Antibody Responses in HBsAg-tg Mice

HBsAg-tg and Ntg mice were repeatedly immunized with surface- and core-based formulations by IN and SC routes simultaneously. Serum conversion was detected in transgenic mice after the 3rd dose but at very low frequency and only for IgG1 subclass, compared with 100% of serum conversion detected in Ntg mice for both IgG subclasses evaluated. The percentages of anti-HBsAg IgG1 and IgG2a serum conversion in HBsAg-tg and Ntg mice are shown in Table 2.

**Table 2:** **Table 2:** Serum conversion to HBsAg in HBsAg-tg mice and in non-transgenic mice

				*Dose 3*						*Dose 5*						*Dose 10*				
*Mouse*		*Isotype*		*Mice^a^*		*Ab (+)*		*%*		*Mice*		*Ab (+)*		*%*		*Mice*		*Ab (+)*		*%*	
Tg		IgG1		11		1		9		8		8		100		4		4		100	
		IgG2a		11		0		0		8		5		63		4		3		75	
Ntg		IgG1		9		9		100		6		6		100		3		3		100	
		IgG2a		9		9		100		6		6		100		3		3		100	

^a^After the 3th, 5th, and 10th doses, three to four mice were sacrificed and spleen cells isolated for T-cells response evaluation

**Graphs 1A and B:**
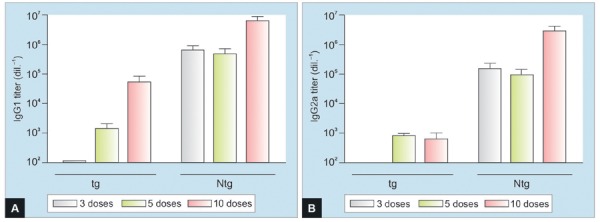
Comparative HBsAg-specific IgG1 (A) and IgG2a (B) antibody levels induced in HBsAg-tg and Ntg mice immunized with formulation containing HBs/HBc antigens, by IN and SC routes. Mice were immunized 10 times at 2-week intervals. Immunized Ntg mice induced higher anti-HBsAg response compared to tg mice (two-tailed Student’s t-test, p < 0.05)

The intensity of anti-HBsAg IgG1 and IgG2a antibody responses induced in HBsAg-tg mice compared with Ntg mice was evaluated, after 3rd, 5^th^, and 10th doses (Graphs 1A and B). The antibody response induced in HBsAg-tg mice is delayed and less intense than that achieved in normal mice (p < 0.05). No antibody response was detected in non-immunized tg and Ntg control mice (Graphs 1A and B).

### Specific T-cell Response is induced in HBsAg-tg Mice

Given the presence of the same genetic background between the Balb/c mice and the HBsAg-tg mice, the dynamic of gamma-IFN secretion by spleen cells from HBsAg-tg and Ntg mice was studied.

**Graph 2:**
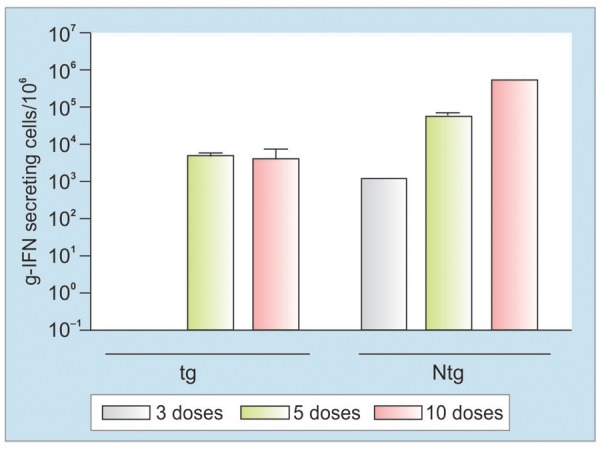
Re-stimulated ELISPOT assays. HBsAg-tg and Ntg mice were immunized with the HBs/HBcAg formulation, by IN and SC routes, every 14 days. Splenocytes were isolated 10 days after the 3rd, 5th, and 10th doses and stimulated *in vitro* with peptide S28-39 of the HBsAg. The frequency of gamma-IFN-secreting cells is represented as the mean value of the number of spot-forming cells per 106 cells ± SD for the three evaluated animals

Splenocytes from Ntg mice stimulated with peptide S_28-39_ generated positive response in 100% immunized mice after the 3th dose; none of the HBsAg-tg mice was positive at this time (Graph 2). The frequency of spleen cells producing gamma-IFN in HBsAg-tg mice was delayed compared to that in Ntg mice, reaching 100% positivity after the 5th administration. The response intensity in HBsAg-tg mice was also lower compared to Ntg mice (Graph 2). However, levels induced after 5th and 10th doses were similar compared to levels reached after the 3rd immunization in Ntg mice, suggesting the immune response was significant even when lower in kinetic of induction comparatively.

### Histopathological Studies

Despite the ability of our immunization strategy to subvert the B- and T-cell tolerance to HBsAg in HBsAg-tg mice, no histological alterations were observed related to the administration of formulations containing HBsAg/HBcAg antigens.

The liver microstructural changes observed for all HBsAg-tg mice used in this work (vaccinated and control) were previously reported.^18^ In summary, there was no evidence of histological damage as a result of an immuno-mediated process.

## DISCUSSION

In the present work, a tg mouse constitutively expressing high levels of HBsAg in serum and different organs was used with the aim of assessing whether simultaneous coadministration of formulations combining HBsAg and HBcAg antigens, by nasal and SC routes, is able to subvert HBsAg immunologic tolerance and cause damage in the organs and tissues where HBsAg is expressed. The previous characterization of these mice demonstrated serum HBsAg expression at high levels during more than 20 months and the production of this antigen in multiple organs.^17,18^ The results presented in this study demonstrate that the administration of at least five doses of the formulations containing HBsAg and HBcAg by the IN and SC routes allowed the detection of humoral and CD8+ cellular responses in all the transgenic animals, as evidenced by ELISA for specific HBsAg antibody and the gamma-IFN ELISPOT (Graphs 1 and 2). Although the response induced in HBsAg-tg mice is delayed and less intense than that achieved in normal mice, it was clear that our immunization strategy allowed tg mice to subvert the T- and B-cell immune-tolerant status, despite the presence of high level of circulating HBsAg. Activation of HBsAg-specific CD8+ T-cells in this system suggests that T-cell tolerance to HBsAg, in our tg model, is also mediated by anergy rather than deletion or exhaustion of HBsAg-specific T-cell clones.^19,20^

Another issue examined in this work was the potential histological change that could be induced as a consequence of the immune responses elicited after active immunization in HBsAg-tg mice.^21^ Histological manifestation of spontaneous, focal liver necrosis, karyomegaly, and some other alterations was observed. These same alterations have been previously reported, during the characterization of the model, by Pérez A et al,^18^ probably related to the accumulation of the highly expressed transgenic product. However, in this study and in their replicate studies, despite the immune response induced at B and T levels, no tissue alterations were detected associated with the vaccination. The study discarded that the absence of hepatic injury could be because the insufficient numbers of CTL cells as the adoptive transfer of immune cells from Ntg Balb/c mice do not result in histopathological damage or altered liver or kidney biochemical markers, suggesting that the immune response induced by the formulation of the present study activates CD8+ non-cytolytic mechanisms preferentially, as has been previously reported.^22^ This might be a limitation later at the clinical trial efficacy studies, however, undoubtedly point to a new proof of the safety of the present formulation, which is also a key issue in the development of a product to subvert the tolerance avoiding prejudicial autoimmune responses. At the end of the day, non-cytolytic mechanisms have also been described to be very efficient in the control of HBV replication.^23^

## CONCLUSION

Repeated immunization with a therapeutic vaccine formulation containing both HBsAg and HBcAg viral antigens induced detectable humoral and cellular immune responses in HBsAg-tg mice. The administration of 10 inoculations using a pharmacologic dose that is much higher than that to be used in humans and a similar treatment schedule of 10 doses every 14 days did not evidence any damage in the main organs or in the immune system organs of the tg mice; besides, the HBsAg is expressed at high levels in the blood, liver, kidneys, and other organs.

Further studies to characterize and optimize the schedule, dose, and formulations, to improve the immune response to such therapeutic vaccine candidate, are need. Finally, the present article proposes the use of tg mice as a tool in the development of therapeutic vaccines where it is required to show that the safety of the product after tolerance subversion and cytolytic and non-cytolytic mechanisms can be involved.
